# Quantitative proteomic analysis using iTRAQ to identify salt-responsive proteins during the germination stage of two *Medicago* species

**DOI:** 10.1038/s41598-018-27935-8

**Published:** 2018-06-22

**Authors:** Ruicai Long, Yanli Gao, Hao Sun, Tiejun Zhang, Xiao Li, Mingna Li, Yan Sun, Junmei Kang, Zhen Wang, Wang Ding, Qingchuan Yang

**Affiliations:** 1 Institute of Animal Sciences, Chinese Academy of Agricultural Sciences, Beijing, 100193 People’s Republic of China; 2 College of Animal Science and Technology, China Agricultural University, Beijing, 100193 People’s Republic of China

## Abstract

Salt stress is one of the primary abiotic stresses responsible for decreasing crop yields worldwide. Germinating seeds can be greatly influenced by saline conditions. In this study, the physiological and phenotypic changes induced by salt treatments (10–50 mM NaCl and Na_2_SO_4_ mixtures) were analysed for Zhongmu-3 (*Medicago sativa*) and R108 (*Medicago truncatula*) seedlings. Our observations indicated that Zhongmu-3 was more salt-tolerant than R108. To characterize the protein expression profiles of these two *Medicago* species in response to salt stress, an iTRAQ-based quantitative proteomic analysis was applied to examine salt-responsive proteins. We identified 254 differentially changed salt-responsive proteins. Compared with control levels, the abundance of 121 proteins increased and 44 proteins decreased in salt-treated Zhongmu-3 seedlings, while 119 proteins increased and 18 proteins decreased in R108 seedlings. Moreover, 48 differentially changed proteins were common to Zhongmu-3 and R108 seedlings. A subsequent functional annotation indicated these proteins influenced diverse processes, such as catalytic activity, binding, and antioxidant activity. Furthermore, the corresponding transcript levels of 15 differentially changed proteins were quantified by qRT-PCR. The data presented herein provide new insights into salt-responsive proteins, with potential implications for enhancing the salt tolerance of *Medicago* species.

## Introduction

Soil salinization is one of the most serious abiotic factors responsible for decreasing crop yields worldwide^1^. Salinization is mainly due to high sodium salt concentrations (NaCl or Na_2_SO_4_), which damage plants *via* osmotic stress, oxidative stress, ion toxicity, nutritional deficiency, and other adverse conditions. In response, plants undergo changes that may ultimately result in death. Salt stress affects diverse physiological and metabolic processes in germinating seeds and in vegetative tissues that decrease growth^2^. According to a report released by the Food and Agriculture Organization of the United Nations (http://www.fao.org/nr/land/information-resources/terrastat/en/), saline soils cover more than 800 million ha, which represents more than 6% of the global land area. The prevalence of salinization and desertification is increasing globally, with more than 10% of the arable land being affected, which has decreased average yields of major crops by more than 50%^3,4^. To maintain growth under salt stress conditions, many plants have evolved defence mechanisms that minimise the entry of salt and limit the cytosolic salt concentration. These defence responses are regulated by a series of plant genes^2^. Seed germination represents one of the most important plant growth stages, and can be greatly influenced by salinity^5^, which prevents or delays germination, decreases seed viability, and leads to secondary dormancy. High salt concentrations may completely inhibit germination, while relatively low salt levels may induce a state of dormancy^6^. Seed germination is controlled by both hormones and environmental factors. Gibberellins, abscisic acid, ethylene and jasmonates play key roles in seed germination^7^. The synthesis of these hormones was also greatly influenced by salt stress^8^.

Previous studies indicated that the molecular mechanism underlying plant salt tolerance is very complex. Numerous genes are affected by salt stress at the transcriptional and translational levels^2,9,10^. Additionally, the expression profiles of many genes involved in responses to salt stress as well as the abundance of the encoded proteins have been characterized in several plant species^11–15^. These studies have included efficient proteomics-based approaches to identify salt-responsive proteins. With the development of mass spectrometers, chromatographic techniques for separating proteins, and bioinformatics tools, advances in proteomics research have increased exponentially over the last few years. The most commonly applied quantitative proteomics methods are two-dimensional gel electrophoresis as well as label-free and isotopic labelling techniques. Stable isotope labelling by amino acids in cell culture (SILAC; metabolic labelling) and isobaric tags for relative and absolute quantitation (iTRAQ; chemical labelling) are two efficient methods for quantifying proteins based on isotope labelling combined with multidimensional liquid chromatography and tandem mass spectrometry (LC-MS/MS)^16–18^. During the past decade, iTRAQ-based proteomics techniques have been widely applied to identify proteins responsive to biotic and abiotic stresses^19–24^. A large number of salt-responsive proteins have been isolated from some plant species by iTRAQ technique in the last few years. Some of these salt-responsive proteins have also been verified to play critical roles in plant salt tolerance^23–27^.

Alfalfa (*Medicago sativa*; tetraploid, 2n = 4× = 32), which is a perennial forage crop, is widely cultivated in many counties for its high yield, good quality, and high protein content. Furthermore, some alfalfa cultivars are highly resistant to salinity, salinity-alkalinity, and drought^2,28,29^. Barrel medic (*Medicago truncatula*; diploid, 2n = 16) is a small annual legume that has been used as a model leguminous organism because it has a small sequenced diploid genome. Additionally, it is self-fertile and amenable to genetic transformation. The *M. truncatula* R108 genotype is more susceptible to salt stress than most alfalfa species. Plants differ considerably in their tolerance to salinity, as reflected by their different growth responses. The majority of these differences are regulated by salt through altered gene expression levels^2,10^. Proteomics-based technologies are powerful tools for studying protein abundance^17,30^. We previously identified several salt-responsive proteins in *M. sativa* and *M. truncatula* roots using two-dimensional gel electrophoresis^31^. In the present study, we completed an iTRAQ-based quantitative proteomic analysis to identify salt-regulated proteins in germinating *M. sativa* and *M. truncatula* seeds and to compare the changes in protein abundance between these two species. The aim of this study is to search salt tolerance candidate proteins from the differentially changed proteins and provide new approaches for alfalfa salt resistance genetically modified breeding in the future.

## Results

### Seed germination and physiological indices

**Figure 1:**
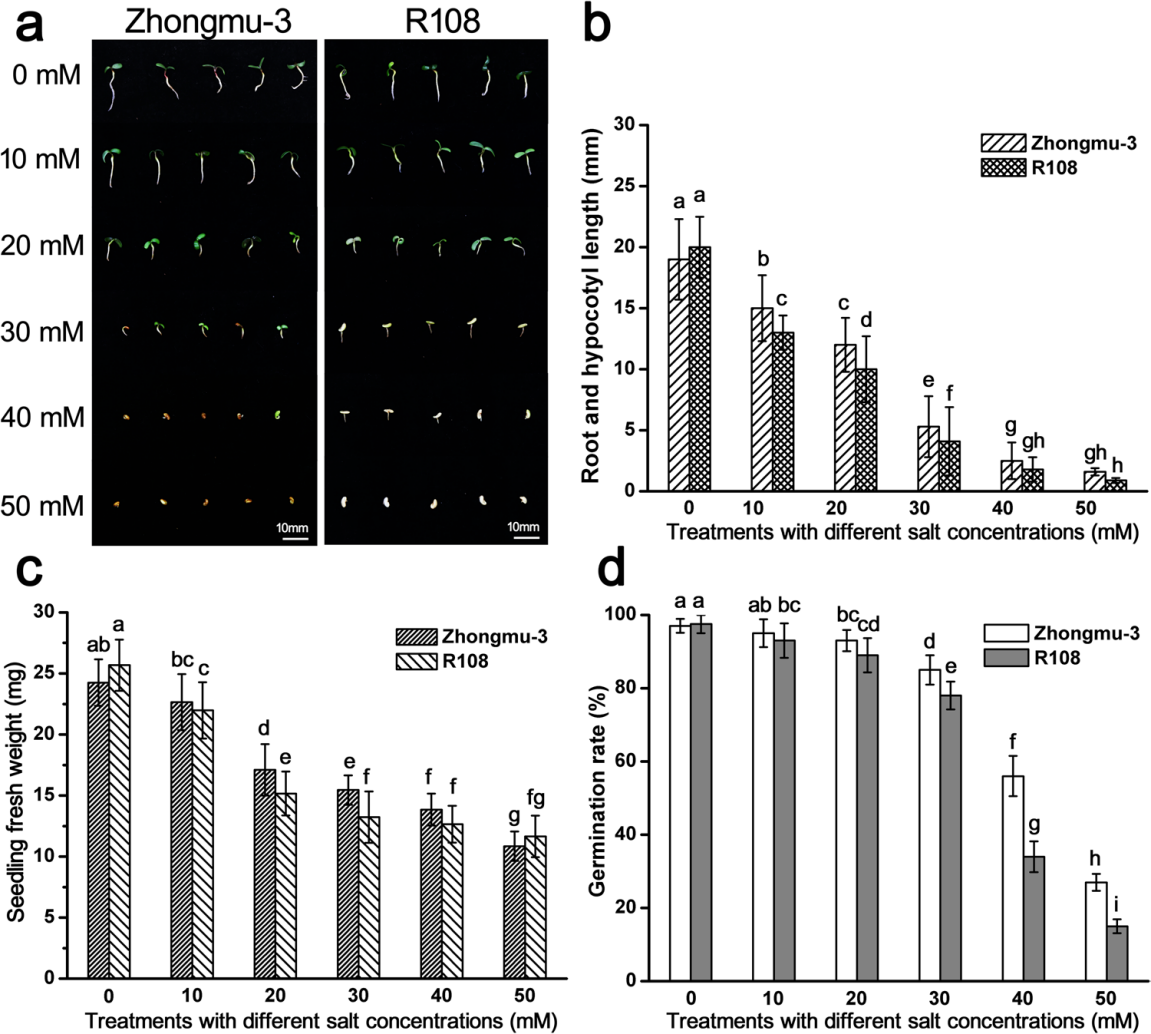
Phenotypes of salt-treated Zhongmu-3 and R108 seedlings. (**a**) Comparison of Zhongmu-3 and R108 seedlings after a 3-day germination in 0–50 mM salt (NaCl and Na_2_SO_4_). (**b**) Root and hypocotyl lengths of Zhongmu-3 and R108 seedlings. (**c**) Average fresh weight of each Zhongmu-3 and R108 seedling. (**d**) Zhongmu-3 and R108 germination rates. The different letters refer to the significant differences at P < 0.05 (Duncan’s multiple range test).

**Figure 2:**
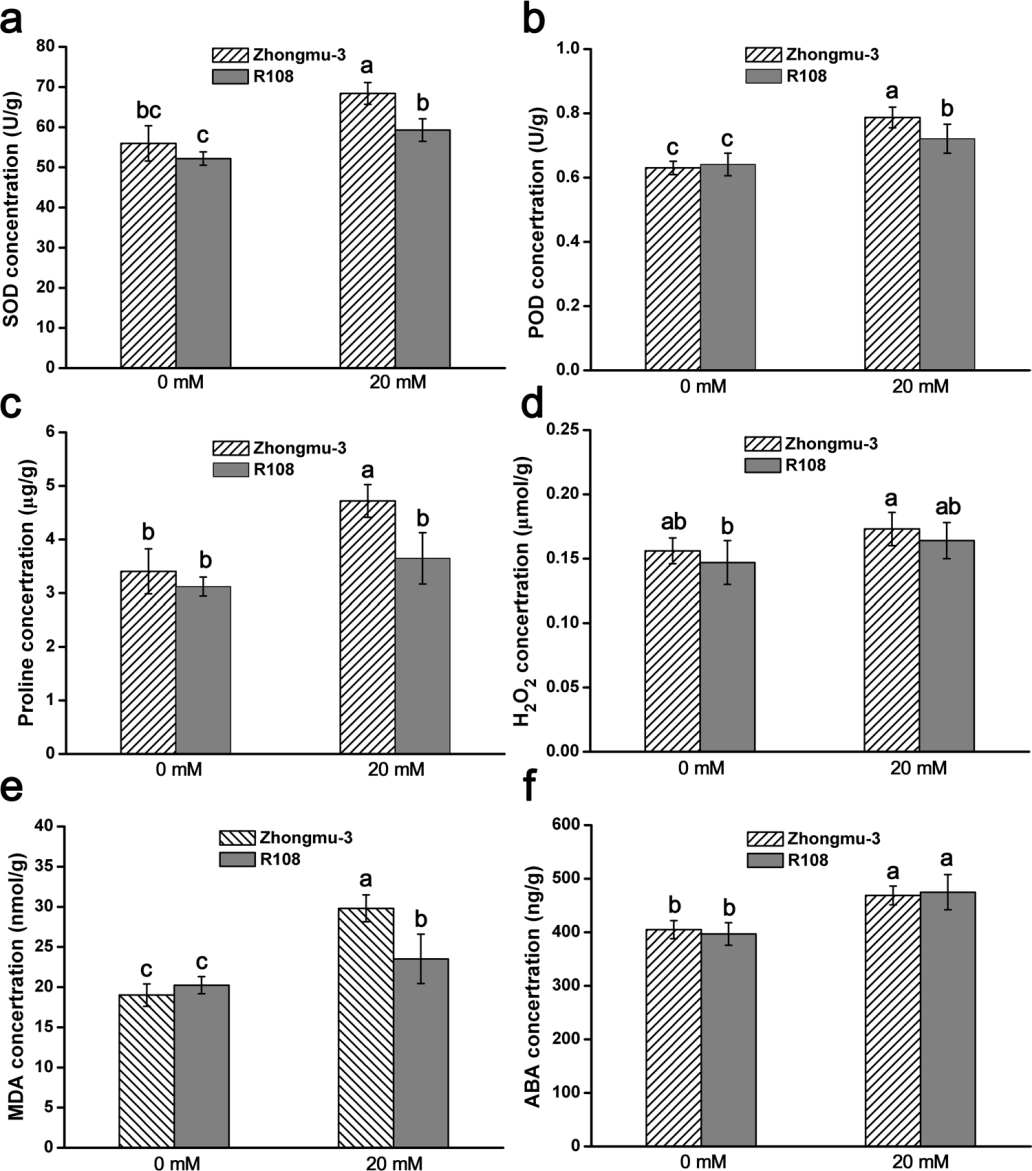
Physiological analysis of Zhongmu-3 and R108 seedlings. (**a**–**f**) Concentrations of SOD, POD, Pro, H_2_O_2_, MDA, and ABA in Zhongmu-3 and R108 seedlings treated with 20 mM salt (NaCl and Na_2_SO_4_). The different letters refer to the significant differences at P < 0.05 (Duncan’s multiple range test).

After a 3-day germination, the growth of the cotyledon, root, and hypocotyl was significantly inhibited with increasing salt concentrations (Fig. 1a,b), resulting in decreased seedling fresh weight (Fig. 1c). Additionally, salt concentrations greater than 20 mM inhibited the germination of Zhongmu-3 and R108 seeds, although R108 seeds were affected more than the Zhongmu-3 seeds (Fig. 1d). At 50 mM salt, only a few seeds were able to germinate. In contrast, the germination rates of the control Zhongmu-3 and R108 seeds were close to 100%. According to the physiological analysis, the concentrations of SOD, POD, Pro, MDA, and ABA in Zhongmu-3 increased significantly in response to salt stress (Fig. 2a–f). Similarly, the concentrations of SOD, POD, MDA, and ABA in R108 increased significantly. The increases of SOD, POD, and Pro were higher in the salt-treated Zhongmu-3 seedlings than in the R108 seedlings. The phenotype, germination rate, and some physiological indices indicated that Zhongmu-3 plants were more salt-tolerant than R108 plants.

### Protein identification

**Figure 3:**
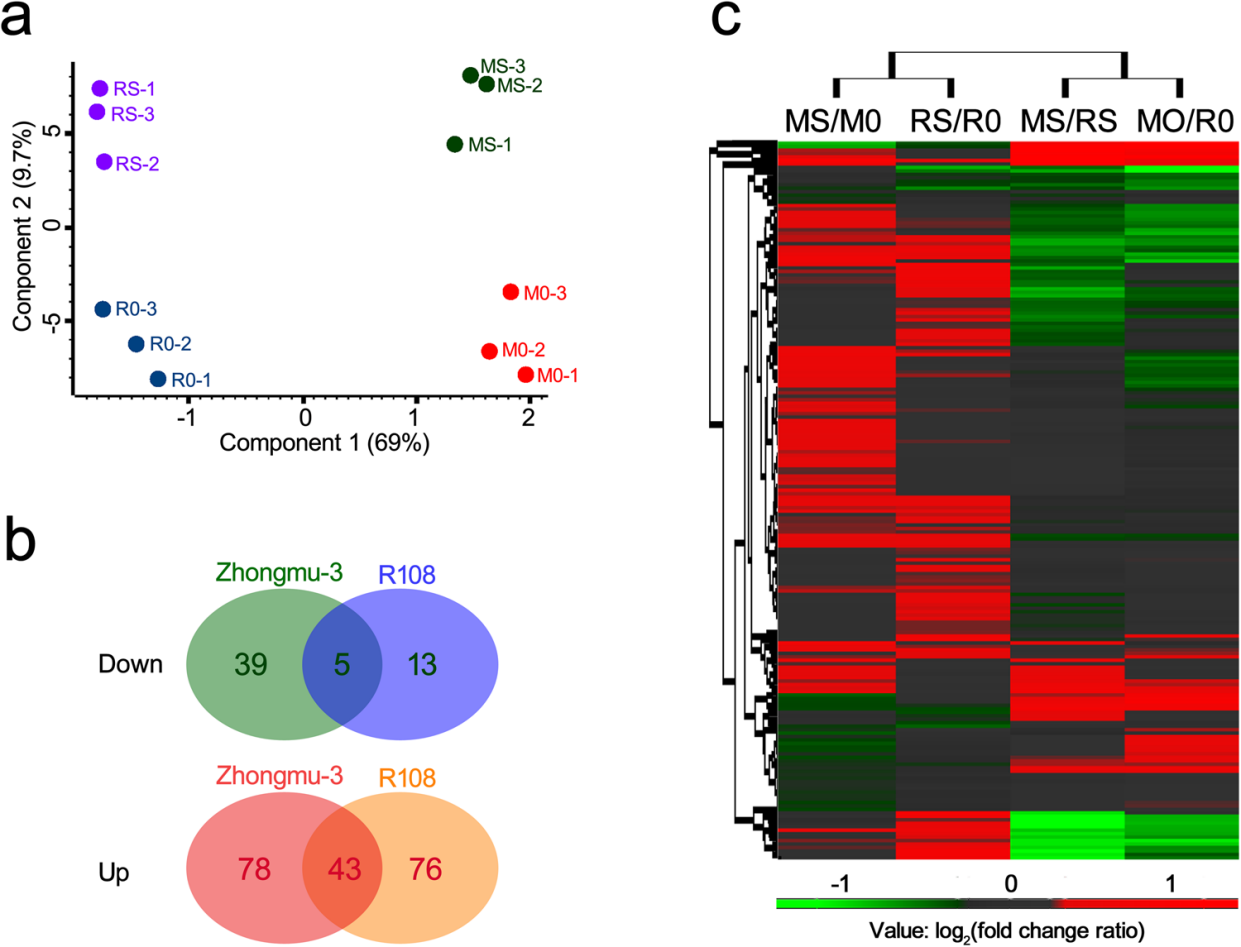
The statistical analysis of iTRAQ data. (**a**) Principal component analysis of the iTRAQ data. MS: Zhongmu-3 seedlings treated with 20 mM salt (NaCl and Na_2_SO_4_); RS: R108 seedlings treated with 20 mM salt (NaCl and Na_2_SO_4_); M0: Zhongmu-3 seedlings without salt treatment; R0: R108 seedlings without salt treatment. Numbers in parentheses represent the percentage of total variance explained by the first and second principal component. (**b**) Venn diagram of differentially changed proteins in Zhongmu-3 and R108 seedlings. (**c**) Hierarchical cluster analysis of the fold change ratio (log_2_ fold change) of the 254 differentially changed proteins identified in Zhongmu-3 and R108 seedlings.

Based on the iTRAQ-LC-MS/MS analysis results, a total of 319,501 spectra were identified for all samples. A total of 4735, 4711 and 4699 proteins were identified from the three replicates respectively. The correlation analysis results showed that the repeatability between the replicates was acceptable (R^2 ^> 0.8, Supplementary Fig. 1). After eliminating low-scoring spectra, a total of 70,122 unique spectra matched to 3,847 proteins at the given thresholds (FDR < 1.0%, probability > 99.0% with at least two identified peptides) in the combined datasets were analysed regarding quantities and functions (Supplementary Tables 1). The principal component analysis (PCA) is a statistical tool for sample classification based on multivariate data. Each point in the PCA graph represents the whole protein profile of one biological replicate sample. Samples with similar behavior in their protein profile are grouped together. In our work the PCA graph for iTRAQ data showed a clear separation between Zhongmu-3 and R108 samples, as well as control and salt-treated samples (Fig. 3a). It also revealed that the iTRAQ data among replicates were consistent. The molecular weights of 93.33% of the identified proteins were between 10 and 110 kDa, and more than 50% of the identified proteins had five or more peptides (Supplementary Table 1).

### Quantification of identified proteins

Salt-responsive proteins were identified based on a comparison of protein abundance between control and salt-treated samples using the iTRAQ data. A fold change ratio >1.20 or <0.83 (p < 0.05) was used to identify differentially changed proteins between the salt-treated and control plants. A total of 165 differentially changed proteins were identified between salt-treated (MS) and control (M0) Zhongmu-3 plants, of which 121 increased and 44 were decreased (Supplementary Table 2). In contrast, 137 differentially changed proteins were identified between salt-treated (RS) and control (R0) R108 plants, of which 119 proteins increased and 18 proteins decreased (Supplementary Table 3). A Venn diagram revealed that 43 increased proteins and five decreased proteins were common to Zhongmu-3 and R108 plants (Fig. 3b). The 254 differentially changed proteins identified in Zhongmu-3 and R108 plants were grouped into several categories based on a hierarchical cluster analysis of the abundance fold change ratios (Fig. 3c).

### Functional annotation of salt-responsive proteins

**Figure 4:**
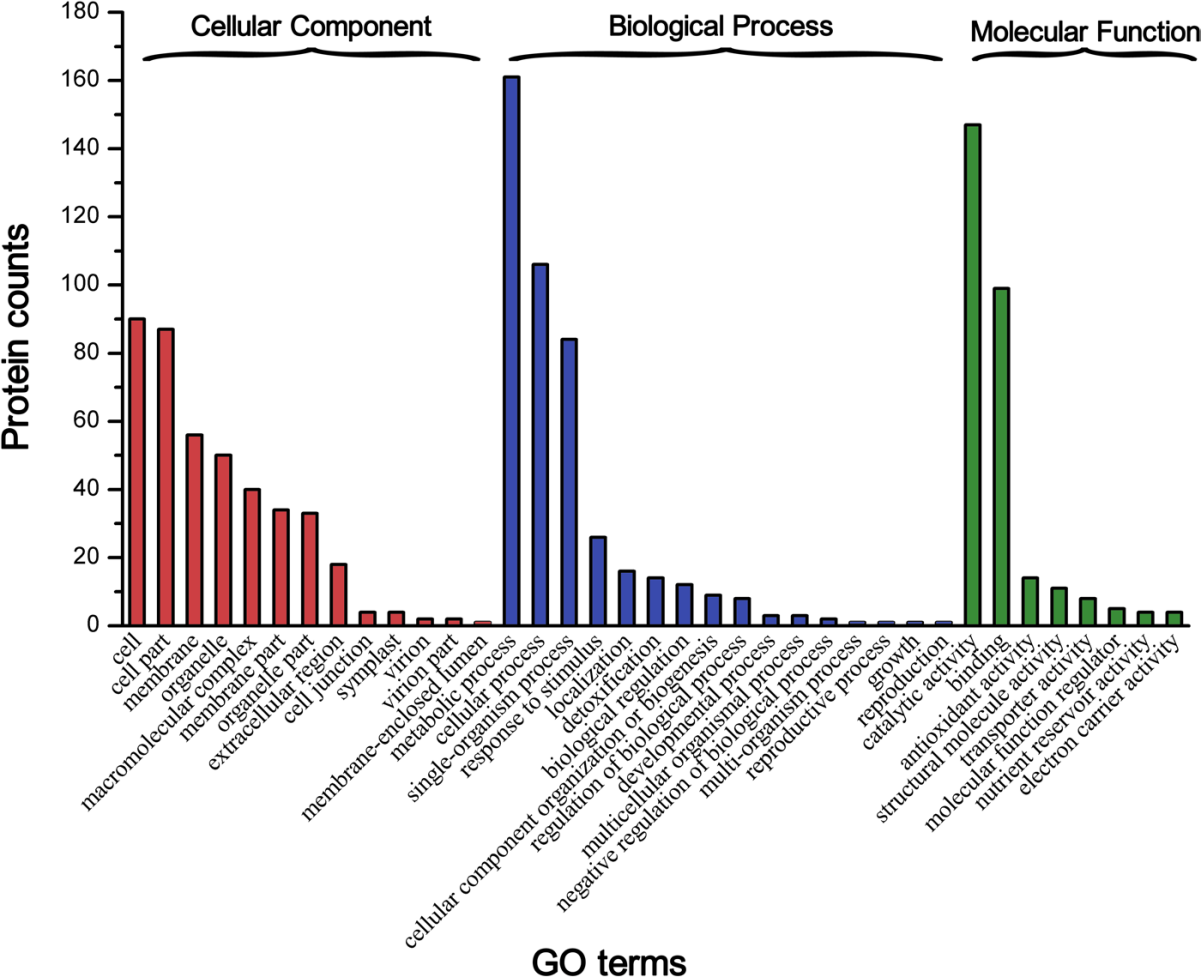
Gene ontology annotations for all differentially changed proteins identified in Zhongmu-3 and R108 seedlings.

To functionally annotate the differentially changed proteins, the amino acid sequences were used as queries in a BLAST search of the NCBI and UniProt databases. According to the GO annotation results, the differentially changed salt-responsive Zhongmu-3 and R108 proteins were classified into eight molecular function categories, 16 biological process categories, and 13 cellular component categories (Fig. 4). The five primary molecular function GO terms were catalytic activity, binding, antioxidant activity, structural molecule activity, and transporter activity. More than 80% of the differentially changed biological process proteins were annotated with metabolic process, cellular process, single-organism process, or response to stimulus GO terms. Meanwhile, cell, cell part, membrane, organelle, macromolecular complex, membrane part, organelle part, and extracellular region were the top eight cellular component GO categories. Furthermore, the GO term enrichment analysis indicated that many proteins were associated with plant abiotic stress response categories, including oxidation–reduction, stimulus response, hydrogen peroxide catabolic process, hydrogen ion transmembrane transport, and ATP synthesis (Supplementary Table 4).

Proteins were also annotated according to the KEGG pathways. Of the 254 differentially changed proteins, 115 were enriched in 67 pathways. Metabolic pathways (75), biosynthesis of secondary metabolites (45), oxidative phosphorylation (15), carbon metabolism (13), photosynthesis (10), phenylpropanoid biosynthesis (12), and ribosome (11) were the seven most enriched KEGG pathways (Supplementary Table 5).

### Analysis of transcripts encoding selected differentially changed proteins

**Figure 5:**
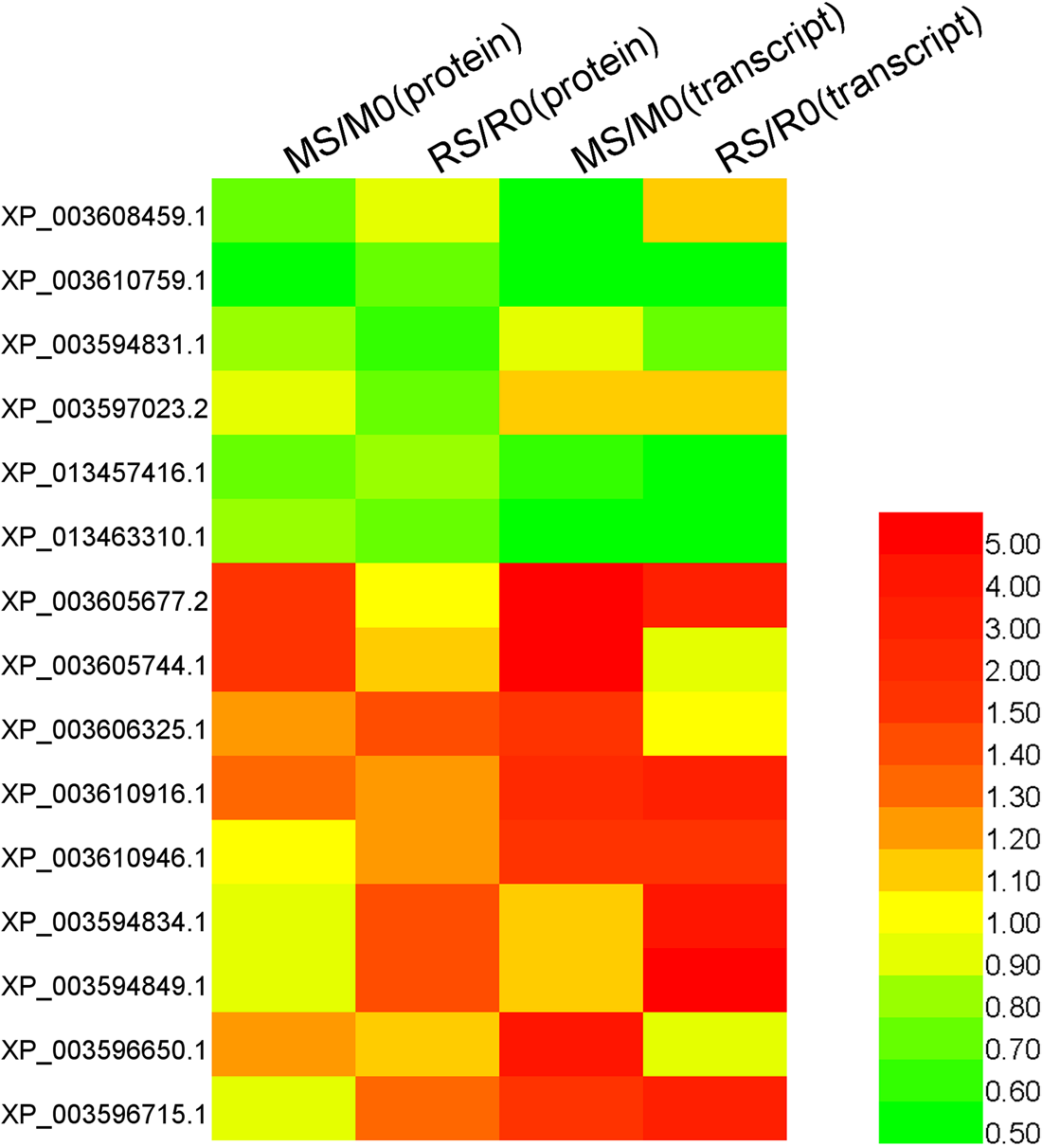
Changes to transcript and protein levels for 15 differentially changed proteins in Zhongmu-3 and R108 seedlings.

To investigate transcription patterns, fifteen identified proteins by iTRAQ underwent qRT-PCR analyses. Five up-regulated proteins, five down-regulated proteins and five proteins with no significant changes in Zhongmu-3 were chosen for verification on mRNA level. Some of these differentially changed proteins have been reported to be involved in stress response^14,15^. The fold changes of protein and transcript abundances are provided in Fig. 5 and Supplementary Table 6. A comparison of the qRT-PCR data for salt-treated and control samples indicated that the transcript abundance fold changes of 11 genes (XP_003608459.1, XP_003610759.1, XP_003594831.1, XP_013457416.1, XP_013463310.1, XP_003610916.1, XP_003594834.1, XP_003594849.1, XP_003596650.1, XP_003597023.2, and XP_003605744.1) were consistent with the protein abundance fold changes. These results suggested that the transcript and protein level changes were similar for most of the analysed proteins.

## Discussion

### Physiological changes

Certain abiotic stresses induce obvious changes to plant physiological parameters. In this study, we observed that exposure to salt stress increased the SOD, POD, Pro, H_2_O_2_, MDA, and ABA concentrations in the seedlings of two *Medicago* species (Fig. 2). Our data indicated that a 20-mM salt treatment leads to oxidative stress and considerably affects the physiological functions of Zhongmu-3 and R108 seedlings. Earlier research confirmed that reactive oxygen species (ROS) are produced during normal metabolic activities, but their abundance increases dramatically in plants exposed to biotic and abiotic stresses^32^. Hydrogen peroxide is a major ROS, while SOD and POD act as antioxidants that protect cellular components from being oxidized by ROS. In response to osmotic stress, plants can accumulate Pro, which functions as an osmoprotectant that prevents the accumulation of ROS in cells^33^. Moreover, MDA, which is a marker of oxidative lipid injuries caused by biotic and abiotic stresses, is mainly generated from peroxides of polyunsaturated fatty acids (tri-unsaturated fatty acids)^34^. A previous study revealed that MDA can affect proteins, membranes, DNA, and specific biomolecules through Schiff’s base addition reactions, and can also function as powerful secondary messengers during responses to abiotic stresses^35^. Furthermore, ABA is involved in many plant developmental processes and is also important for signalling during plant responses to environmental stresses and plant pathogens. In this study, compared with the control levels, the increases in SOD, POD, Pro, and MDA concentrations were higher in the salt-treated Zhongmu-3 seedlings than in the R108 seedlings. In contrast, there were no significant differences in the increases in H_2_O_2_ and ABA concentrations between Zhongmu-3 and R108 seedlings. These results imply that the salt tolerance and regulatory mechanisms underlying responses to salt stress differ between Zhongmu-3 and R108 plants. Our iTRAQ results verified this hypothesis. We detected 22.44% more salt-responsive differentially changed proteins in Zhongmu-3 seedlings (165) than in R108 seedlings (137). Additionally, there were only 48 differentially changed proteins common to both Zhongmu-3 and R108 plants (Fig. 3b).

### Antioxidant responses

To decrease the cellular damage caused by ROS, plants produce antioxidants, including enzymes, that scavenge ROS. According to our iTRAQ data, there was a slight increase in the abundance of one SOD (XP_003618947.1) (i.e., 1.11- and 1.18-fold changes in Zhongmu-3 and R108, respectively) in response to the salt treatment. In contrast, there were no changes to the abundance of two other SODs (XP_013461533.1 and XP_003608775.1), while there was a slight decrease in the XP_013467307.1 level (i.e., 0.89- and 0.81-fold changes in Zhongmu-3 and R108, respectively) in salt-treated seedlings. Additionally, nine PODs were identified by iTRAQ, including two (XP_003608471.1 and XP_003609961.2) that increased in salt-treated Zhongmu-3 seedlings, while six (XP_003609961.2, XP_003596715.1, XP_013452025.1, XP_003602461.1, XP_003594487.1, and XP_003612078.1) increased in salt-treated R108 seedlings. Proline is also an important antioxidant for protecting cells under stress conditions^33^. In stressed plants, delta-1-pyrroline-5-carboxylate synthetase (P5CS) is the rate-limiting enzyme during Pro biosynthesis^36^. Our iTRAQ results revealed that P5CS (XP_013448975.1) increased by more than 1.20-fold (for its p value > 0.05, it was not listed in Supplementary Table 3) in salt-stressed R108 seedlings, but was unaffected in salt-treated Zhongmu-3 seedlings (Supplementary Table 1). The GO term enrichment analysis confirmed that 14, 50, and 10 differentially changed proteins were included in the antioxidant activity, response to oxidative stress, and reactive oxygen species metabolic process categories, respectively (Supplementary Table 4). These results imply that the mechanism responsible for the biosynthesis and regulation of antioxidants is complex and differs between Zhongmu-3 and R108 plants.

### Proteins associated with salt tolerance

In addition to antioxidants, other proteins influencing salt tolerance were identified in Zhongmu-3 and/or R108 seedlings. According to the GO annotations, 28, 10, and 9 differentially changed proteins were grouped in the response to abiotic stimulus, osmotic stress, and salt stress categories, respectively. These proteins included H^+^-ATPase, heat shock protein (HSP), annexin, and leucine-rich repeat (LRR) receptor-like kinase, which are crucial for regulating salt stress responses. An earlier study concluded that tobacco salt tolerance is enhanced by the overexpression of the vacuolar-type H^+^-ATPase^37^. We observed that the abundance of a vacuolar-type H^+^-ATPase subunit protein (XP_003611036.1) increased 1.51- and 1.15-fold in salt-treated Zhongmu-3 and R108 seedlings, respectively. The HSPs form a family of proteins that function as molecular chaperones that help to refold proteins and prevent the aggregation of proteins in response to stresses^38^. Our data indicated that HSP40 (XP_003601936.1) abundance increased in Zhongmu-3 and R108 seedlings in response to salt stress. Annexin, which refers to a group of proteins associated with various cellular and physiological processes, reportedly helps to protect plants from biotic and abiotic stresses (e.g., salt and drought stress)^39–41^. Two annexins (XP_013445083.1, XP_003615096.1) increased significantly in salt treatments in the current study. Meanwhile, LRR receptor-like kinase is important for diverse biological pathways, including the stress response pathway^42–44^. We revealed that the abundance of one LRR receptor-like kinase (XP_003597454.1) increased more than 1.4-fold in salt-treated Zhongmu-3 and R108 seedlings. The abundance of another LRR receptor-like kinase increased significantly in R108 plants, but not in Zhongmu-3 plants. The abundance of these salt-responsive proteins may enhance the tolerance of Zhongmu-3 and R108 plants to salt stress.

### Hormone-related proteins involved in salt stress responses

**Figure 6:**
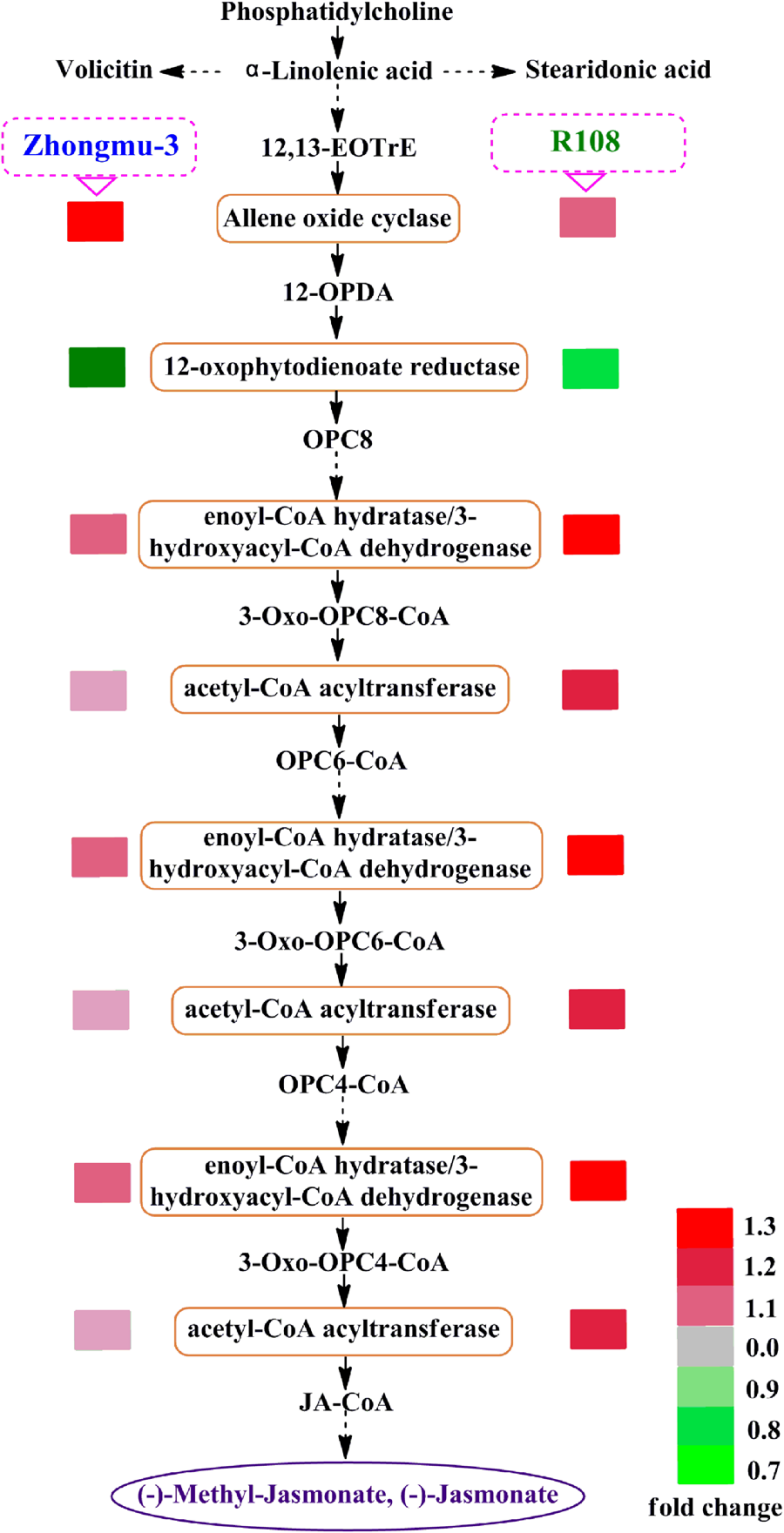
Differentially changed proteins associated with alpha-linolenic acid (jasmonic acid) metabolism. Different colours reflect the changes in protein abundance based on the iTRAQ data.

Hormones are critical for plant growth and development as well as for stress responses. Specifically, jasmonic acid (JA), ABA, and melatonin are important for responses to biotic and abiotic stresses^8,45–47^. These stresses induce plants to increase the production of ABA^8^. We observed that the abundance of some ABA-responsive proteins, including XP_003594849.1 and XP_003608459.1, increased in salt-treated Zhongmu-3 and R108 seedlings. In addition to their effects on plant development, JAs often function as vital signalling compounds in response to biotic and abiotic stresses^45,46^. Our data indicated that five differentially changed proteins were related to the alpha-linolenic acid metabolic pathway, which is the main JA biosynthesis pathway. Most of these proteins increased in salt-stressed seedlings (Fig. 6), which is consistent with the findings of a previous study that concluded methyl JA counteracts the negative effects of salt stress on plant growth^48^. Moreover, melatonin is a small indole molecule that was initially observed in animals, in which it is produced in the pineal gland. However, plants also produce melatonin as an antioxidant to mitigate the adverse effects of oxidative stress^47^. Caffeic acid O-methyltransferase (CAMT) is a key enzyme involved in the last two steps of the melatonin biosynthesis pathway^49^. The abundance of two CAMTs (XP_003605744.1 and XP_003618025.2) was observed to increase considerably in salt-treated Zhongmu-3 and R108 seedlings (Supplementary Table 1), implying melatonin is important for the elimination of ROS under salt stress conditions.

### Cell wall metabolism

Plant cell walls, which are composed of cellulose, hemicellulose, lignin, pectin, and structural proteins, are critical for maintaining cell shapes and providing mechanical strength to withstand turgor pressure^50^. Abiotic stresses often decrease plant shoot growth and damage cell wall structures and the associated metabolic activities. We identified 12 differentially changed proteins related to phenylpropanoid biosynthesis, which is involved in lignin production (Supplementary Fig. 2). The abundance of most of these proteins, such as caffeoyl-CoA 3-O-methyltransferase (XP_003620871.1) and caffeic acid O-methyltransferase (XP_003618025.2) increased in response to salt stress conditions, which is consistent with our previous studies that concluded caffeoyl-CoA 3-O-methyltransferase levels in alfalfa and barrel medic seedlings increase after exposures to high salt concentrations^31,51^.

## Conclusions

Our analyses of the physiological characteristics and phenotypes induced by salt stress suggest that Zhongmu-3 and R108 germination and growth are inhibited by salt (NaCl and Na_2_SO_4_) concentrations greater than 20 mM. However, Zhongmu-3 seedlings are considerably more salt-tolerant than R108 seedlings. We also identified 254 salt-responsive proteins from Zhongmu-3 and R108 using an iTRAQ-based method, including 48 differentially changed proteins common to both *Medicago* species. Functional annotations revealed the analysed stress-responsive proteins exhibit diverse molecular functions affecting various cellular processes, including catalytic activity, binding, antioxidant activity, structural molecule activity, and transporter activity. Some of these salt-responsive proteins were confirmed or predicted to be critical for regulating salt stress responses. The observed changes to protein abundance profiles provide new insights into the regulatory mechanism responsible for salt stress responses in leguminous plants and may serve as the basis for future studies aimed at enhancing the salt tolerance of alfalfa and other plants.

## Methods

### Plant growth condition and treatment

Alfalfa (*M. sativa* cv. Zhongmu-3) and barrel medic (*M. truncatula* R108) were analysed. Zhongmu-3 alfalfa is saline-tolerant and widely cultivated in the North China Plain, especially on the arable land at low to moderate elevations with saline and alkaline soils. Zhongmu-3 and R108 seeds were surface-sterilized in 75% ethanol for 10 min and then washed three times with double-distilled water. Approximately 100 seeds were germinated in a Petri dish (diameter: 15 cm) containing one sheet of Whatman filter paper moistened with 10 mL 10, 20, 30, 40, or 50 mM salt solution (NaCl:Na_2_SO_4_ = 1:1). Seeds germinated in double-distilled water were used as controls. All seeds were placed in a growth chamber at 25 °C under long-day conditions (16-h light/8-h dark) and the germination rate was calculated for three biological replicates after 3 days. In this study, MS and M0 represent the Zhongmu-3 alfalfa samples treated with 20 mM salt and control conditions, respectively, while RS and R0 refer to the barrel medic R108 samples exposed to 20 mM salt and control conditions, respectively.

### Phenotypic and physiological analyses

After the 3-day germination, the phenotype and physiological characteristics of the seedlings treated with 20 mM NaCl and Na_2_SO_4_ were compared with those of the controls. Root and hypocotyl lengths were measured for five seedlings per biological replicate, while fresh weights were determined for 10 seedlings per biological replicate. The analysed samples were then ground to a fine powder in liquid nitrogen using a mortar and pestle. The samples were subsequently examined in an enzyme-linked immunosorbent assay (Nanjing Jiancheng Bioengineering, China) using the RT-6100 Microplate Reader (Rayto, USA) to measure the concentrations of superoxide dismutase (SOD), peroxidase (POD), proline (Pro), hydrogen peroxide (H_2_O_2_), malondialdehyde (MDA), and abscisic acid (ABA).

### Protein extraction and digestion

For each of three biological replicates of the control and treated seedlings, approximately 1 g 3-day-old seedlings was frozen in liquid nitrogen and ground with a mortar and pestle (i.e., 12 samples in total). The ground samples were mixed with lysis buffer [1 mL/0.1 g; 7 M urea, 2 M thiourea, 4% CHAPS, and 1% Protease Inhibitor Cocktail (Roche, USA)] in centrifuge tubes. The solutions underwent an ultrasonication and were then incubated at room temperature for 30 min before being centrifuged at 15,000 × g for 1 h at 4 °C. The supernatants were collected, precipitated in four volumes of 10% (w/v) trichloroacetic acid/acetone solution, and stored at −20 °C overnight^52^. The samples were centrifuged at 15,000 × g for 10 min at 4 °C and the pellets were rinsed three times with ice-cold acetone. The washed pellets were air-dried, dissolved in lysis buffer, and analysed with a Bradford assay to measure the protein concentration^53^. For each sample, the protein (200 μg) dissolved in lysis buffer was reduced with 25 mM DTT, alkylated with 50 mM iodoacetamide, and subjected to centrifugal ultrafiltration (12,000 × g for 20 min) through a 10-kDa cut-off filter (Millipore, USA). The filters were washed three times with 100 μL dissolution buffer (20 mM triethylammonium bicarbonate), followed by a centrifugation at 12,000 × g for 20 min. A 50-μL aliquot of dissolution buffer (20 mM triethylammonium bicarbonate) containing 4 μg trypsin was added to each filter, which was then incubated at 37 °C for 12 h (filter aided sample preparation method)^54^. The peptides were collected by a centrifugation at 12,000 × g for 10 min. Finally, the filters were washed with 50 μL dissolution buffer (20 mM triethylammonium bicarbonate). The collected peptide-containing dissolution buffers were combined.

### iTRAQ labelling and LC-MS/MS proteomic analysis

Peptides digested from 100 μg proteinfor each sample in dissolution buffer was labelled with isobaric tags from the iTRAQ Reagent-8plex Multiplex Kit (AB Sciex, USA) according to the manufacturer’s recommended procedure (114 for three biological replicates of R0, 115 for three biological replicates of RS, 117 for three biological replicates of M0, and 118 for three biological replicates of MS, 119 for the pool of R0, RS, M0 and MS). The labelled peptide mixtures were then pooled and dried by vacuum centrifugation. The labelled peptides were then fractionated using a strong cation exchange PolySulfoethyl column (Durashell-C18; 4.6 mm × 250 mm, 5 μm, 100 Å; Agela, USA) in an HPLC system (RIGOL L-3000, China) at a flow rate of 0.7 mL/min. Retained peptides were eluted using Buffer A (2% acetonitrile alkalized to a pH of 10 with ammonia water) and Buffer B (98% acetonitrile alkalized to a pH of 10 with ammonia water). The following chromatographic gradients were applied: 5 min, 5–8% Buffer B; 30 min, 8–18% Buffer B; 27 min, 18–32% Buffer B; 2 min, 32–95% Buffer B; 4 min, 95% Buffer B; 4 min, 95-5% Buffer B. The eluant was monitored based on an absorbance at 214 nm. The collected fractions were pooled to obtain 10 final fractions. Each fraction was concentrated by vacuum centrifugation and reconstituted in 20 μL 2% methanol (v/v) and 0.1% formic acid (v/v). The fractions were analysed with a Nano HPLC system (Thermo, USA) coupled to a Q-Exactive mass spectrometer (Thermo, USA). Sample (10 μL) from each fraction was injected 3 times to the Nano HPLC system. Peptides were separated on a C18 analytical reverse phase column (75 μm × 12 cm, 3 μm, 200 Å, Thermo, USA) at a flow rate of 350 nL/min and a linear LC gradient profile was used to elute peptides from the column. Then the fractions were analyzed by Q-Exactive mass spectrometer (Thermo, USA). Data were acquired using a data-dependent acquisition mode. The 10 most abundant multiply-charged peptides with an m/z between 300 and 1800 were selected for MS/MS with 15 s dynamic exclusion setting.

### Protein identification and quantification

The raw mass data were used to search the UniProt *M. truncatula* database (57,693 entries) with the Mascot server (version 2.6; Matrix Science, UK), assuming the proteins were digested by trypsin. The Mascot search was completed with a fragment ion mass tolerance of 0.020 Da and a parent ion tolerance of 10.0 ppm. Additionally, carbamidomethyl modifications of cysteine residues as well as iTRAQ 8-plex modifications of lysine residues and at the N-terminus were specified as fixed modifications. Meanwhile, the Scaffold Q+ program (version 4.6.2; Proteome Software Inc., USA) was used to identify and quantify proteins. Peptides were considered to be correctly identified if they could be established at greater than 99.0% probability to achieve a false discovery rate (FDR) less than 1.0% according to the Scaffold Local FDR algorithm. Proteins were considered to be correctly identified if they could be established at greater than 99.0% probability to achieve a false discovery rate (FDR) less than 1.0% with at least two identified peptides. Data was normalized iteratively on intensities (across samples and spectra) according to the method reported by Oberg *et al*.^55^. Final protein abundances were based on the medians of three biological replicates. Spectra data were log-transformed, pruned of those matched to multiple proteins and those missing a reference value, and weighted by an adaptive intensity weighting algorithm. Additionally, the fold change ratios of the identified proteins between two samples were used to assess the significance of any changes, while significant differences (p < 0.05) between the means of two samples were based on Student’s *t*-test. Differentially changed proteins were identified as proteins with a fold change ratio >1.20 or <0.83 (p < 0.05)^20,56^.

### Functional annotation and classification

Differentially changed proteins were functionally annotated based on gene ontology (GO) classifications using the Blast2GO program (https://www.blast2go.com/). Furthermore, the KOBAS 3.0 online program (http://kobas.cbi.pku.edu.cn/) was used to determine which Kyoto Encyclopedia of Genes and Genomes (KEGG) pathways were enriched among the salt-responsive proteins^57–59^.

### RNA extraction and quantitative real-time PCR

Total RNA was extracted from seedlings using Trizol (Invitrogen, USA) and then dissolved in RNase-free double-distilled H_2_O. The RNA quality was analysed with a NanoDrop 2000 spectrophotometer (Thermo, USA), after which cDNA was synthesized using the PrimeScript Reverse Transcriptase Kit (Takara, Japan) for a subsequent quantitative real-time polymerase chain reaction (qRT-PCR). The qRT-PCR was completed using the ABI 7500 Real-Time PCR system (Applied Biosystems, USA). The *β-actin* gene served as a control to normalize target gene quantities. The gene-specific qRT-PCR primers are listed in Supplementary Table 7. The PCR program (i.e., 40 cycles of 95 °C for 15 s and 60 °C for 30 s) was followed by a melting curve analysis. The transcript abundance for each gene was normalized to that of *β-actin*. The relative expression level was calculated as follows: ratio = 2^−ΔΔCt^ = 2^−(ΔCtt–ΔCtc)^ (Ct: cycle threshold; Ctt: Ct of the target gene; Ctc: Ct of the control gene)^60^. Each measurement was completed using three biological replicates.

### Statistical analysis

All experiments were conducted with three biological replicates. Student’s *t*-test, a principal component analysis (PCA), correlation analysis and hierarchical cluster analyses were completed using the Perseus program (version 1.6; Germany)^61^. Venn diagram analyses involved an online tool (http://jvenn.toulouse.inra.fr/app/index.html). For the phenotypic and physiological analyses, the mean differences were compared using Duncan’s multiple range test (p < 0.05) to determine significance. The values provided in the figures are the means ± standard errors.

## Electronic supplementary material

Supplementary information

Supplementary Table 1

Supplementary Table 2

Supplementary Table 3

Supplementary Table 4

Supplementary Table 5

Supplementary Table 6

Supplementary Table 7
