# Supplementary material for: Quantitative proteomic analysis using iTRAQ to identify salt-responsive proteins during the germination stage of two Medicago species
Source: Sci Rep. 2018 Jun 22;8:9553. doi: 10.1038/s41598-018-27935-8 (PMC6015060; doi:10.1038/s41598-018-27935-8)
Supplement: Supplementary file 1 — Supplementary information [file 41598_2018_27935_MOESM1_ESM.docx]

**Quantitative proteomic analysis using iTRAQ to identify salt-responsive proteins during the germination stage of two *Medicago* species**

Ruicai Long^1,*^, Yanli Gao^1,*^, Hao Sun^1^, Tiejun Zhang^1^, Xiao Li^1^, Mingna Li^2^, Yan Sun^2^, Junmei Kang^1^, Zhen Wang^1^, Wang Ding^1^, Qingchuan Yang^1^

^1^ Institute of Animal Sciences, Chinese Academy of Agricultural Sciences, Beijing 100193, People’s Republic of China.

^2^ College of Animal Science and Technology, China Agricultural University, Beijing 100193, People’s Republic of China.

**Supplementary information**


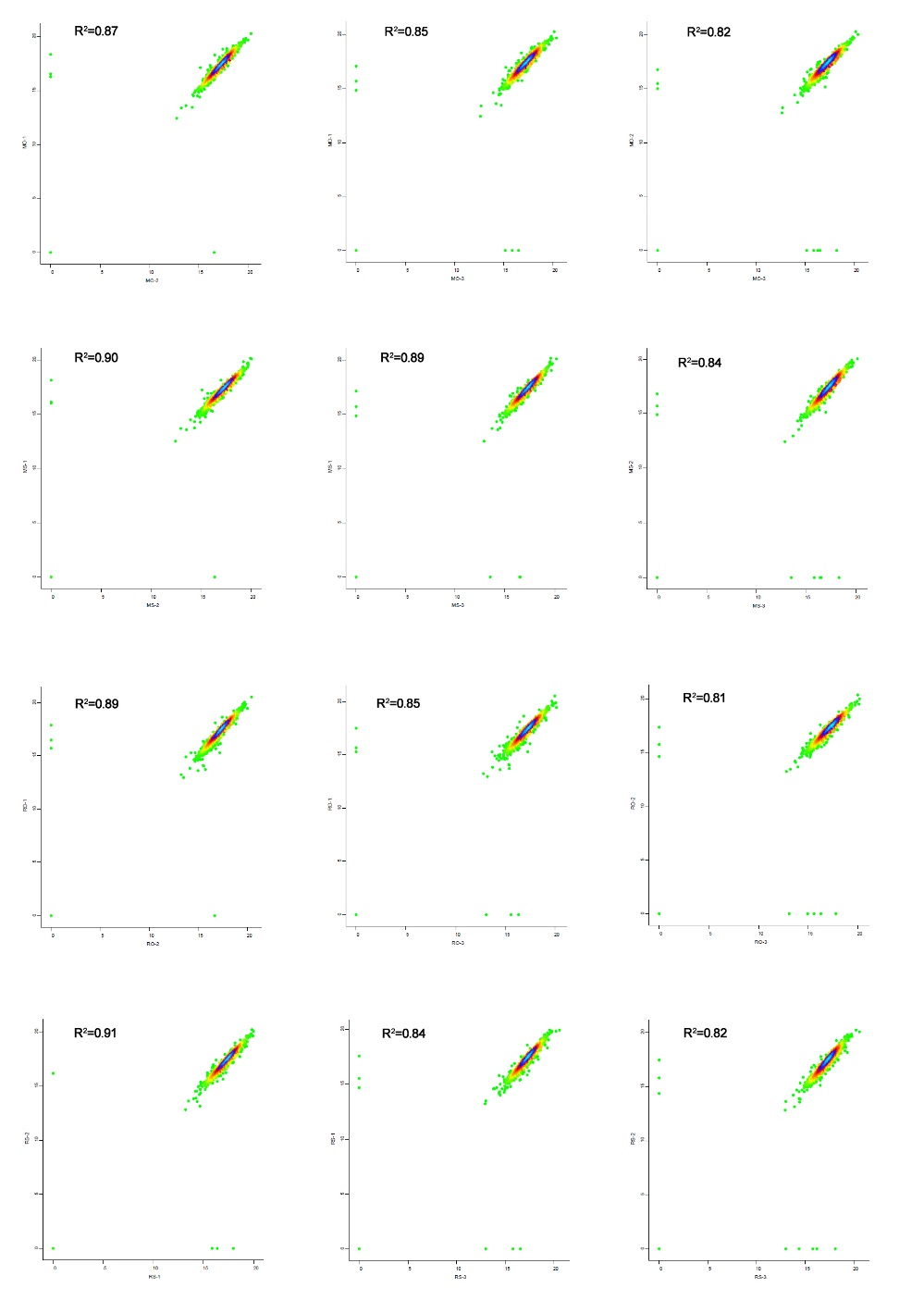


**Supplementary Fig. 1** The correlation analysis of iTRAQ replicates.


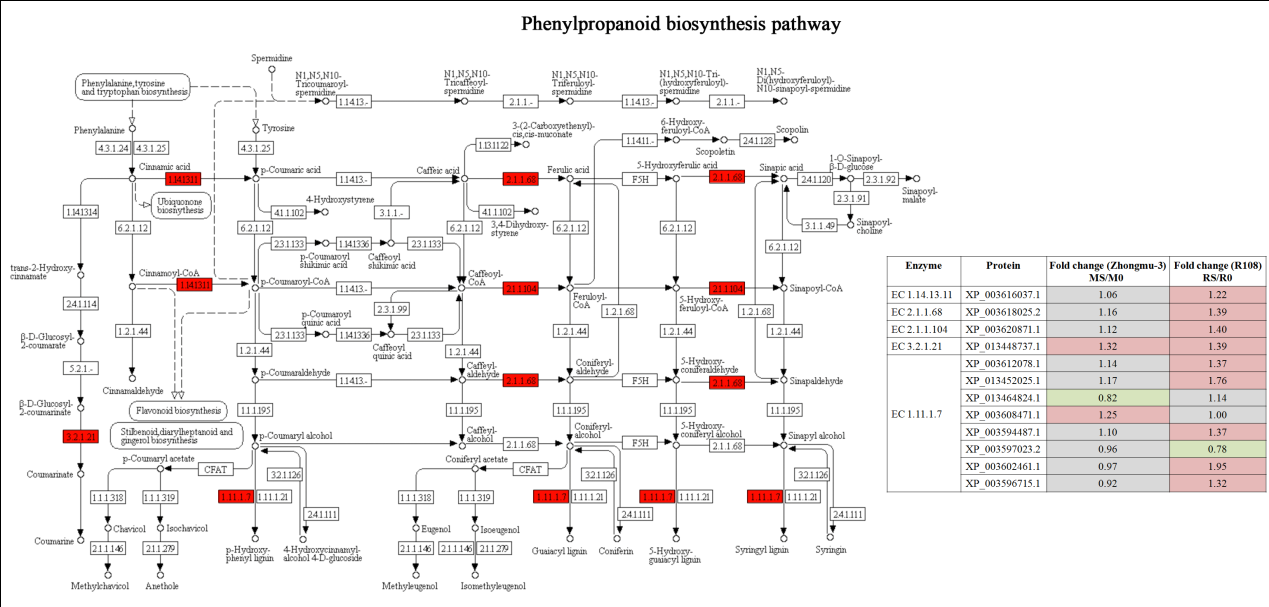


**Supplementary Fig. 2** Differentially changed proteins involved in the phenylpropanoid biosynthesis pathway affecting lignin biosynthesis ^57-59^.

**Supplementary Table 1** Zhongmu-3 and R108 proteins identified by iTRAQ.

**Supplementary Table 2** Differentially changed proteins in Zhongmu-3 seedlings (fold change ratio > 1.20 or < 0.83).

**Supplementary Table 3** Differentially changed proteins in R108 seedlings (fold change ratio > 1.20 or < 0.83).

**Supplementary Table 4** Gene ontology classifications of differentially changed proteins and the results of an enrichment analysis.

**Supplementary Table 5** KEGG pathways associated with the identified differentially changed proteins.

**Supplementary Table 6** The fold changes of protein and transcript abundances of 15 identified proteins by iTRAQ.

**Supplementary Table 7** Details regarding the qRT-PCR primers used in this study.
